# Long-term Developmental Effects of Lactational Exposure to Lead Acetate on Ovary in Offspring Wistar Rats 

**Published:** 2011-03-21

**Authors:** Mehran Dorostghoal, Ahmad Ali Moazedi, Mehrnaz Moattari

**Affiliations:** Biology Department, Faculty of Sciences, Shahid Chamran University of Ahwaz, Ahwaz, Iran

**Keywords:** Ovarian Follicles, Development, Fertility, Lead Acetate

## Abstract

**Background:**

During the last decades, environmental contamination by lead generated from human activities has become an evident concern. The present study assessed the long-term effects of neonatal exposure to different doses of lead acetate on the ovaries of offspring rats.

**Materials and Methods:**

Pregnant female Wistar rats were randomly divided into a control and three experimental groups. The experimental groups received 20, 100 and 300 mg/L/day lead acetate via drinking water during lactation. Ovaries of the offspring were removed at 30, 60, 90 and 120 days of age, their weights recorded and fixed in Bouin’s solution. Following tissue processing, 5 μm serial sections were stained with hematoxylin-eosin, and then, the numbers and diameters of ovarian follicles and corpora lutea were estimated.

**Results:**

Ovary weights decreased significantly (p<0.05) in the 300 mg/L/day dose groups at 30, 60 and 90 days postnatal development. Significant dose-related decreases were seen in the numbers of primary, secondary and antral follicles in 100 (p<0.05) and 300 mg/L/day doses groups at 30 and 60 days of age (p<0.01). There was significant decrease in mean number of corpora lutea in the 100 (p<0.05) and 300 (p<0.01) mg/L/day dose groups at 60 days of age. It seems that neonatal lead treatment has transient effects on follicular development in the ovary of offspring and ovarian parameters gradually improve until 90 days of age.

**Conclusion:**

The present study showed that maternal lead acetate exposure affects prepubertal ovarian follicle development in a dose dependent manner, but ovarian parameters gradually improve during the postpubertal period.

## Introduction

The female reproductive system and, therefore human fertility may be affected by exposure to environmental toxicants. In this regard, most attention has been paid to toxic environmental factors that cause ovarian toxicity (1). Epidemiological and animal studies have shown that trace metals such as lead, cadmium and mercury have the potential to disrupt ovarian function (2).

Lead is a ubiquitous environmental pollutant widely dispersed in the environment and remains in the biotope. Exposure to lead may be via contaminated food or water and fuel additives (3). Reports state that paint, gasoline, printing material and acid batteries (4), as well as some industries such as mining and the refining of plants (5) are the greatest sources of exposure to lead. It is well known that lead passes through the placenta from mother to fetus and accumulates in fetal tissues during gestation (6) and can be obtained through the milk during lactation (7).

Golmohammadi et al. found an association between mean concentrations in blood lead of mothers and newborns (8). Since gastrointestinal absorption may be increased during lactation, along with increased calcium absorption, practically all lead ingested in contaminated milk is absorbed by pups. However, it has been shown that increases in maternal blood lead levels during gestation do not affect the birth weight of neonates (9).

Lead can be concentrated in the cell nucleus, thus perturbing cell proliferation and DNA synthesis (10, 11). It is reported that the female gamete physiology in vitro is modified by exposure to very low levels of lead (12). Specific effects of lead on ovarian function have been observed in mice (5, 13), rats and monkeys (14-16). Longer and more variable menstrual cycles have been found in lead treated female Rhesus monkeys (17). Moreover, it has been shown that circulating levels of both luteinizing hormone (LH) and estradiol (E2) decline in prepubertal females exposed maternally to low levels of lead (4, 18, 19). In contrast, other investigators have detected little or no reproductive toxicity in adults when exposures were restricted to early developmental periods (20-22). The current study evaluates long-term effects of lactational exposure to different doses of lead acetate on ovarian development in offspring Wistar rats.

## Materials and Methods

### Animals and treatments

The Ethics Committee of Shahid Chamran University of Ahwaz approved this research project. Forty female Wistar rats were obtained from the animal house of the Jundishapour Medical Sciences University of Ahwaz and kept under specific conditions on a constant 12-hour light/dark cycle and at a controlled temperature of 22 ± 2°C. All rats had unlimited access to standard pellet food (Pars Co.) and distilled water. After acclimatizing to the laboratory conditions for one week, female Wistar rats (100 ± 10 days old) were mated overnight at a proportion of three females per male. After childbirth, mothers and their pups were randomly divided into four equal groups: control and three treatment groups that received 20, 100 and 300 mg/L/day lead acetate (Merk Co.) in drinking water from day 1 to day 21 of the lactational period. Doses were established from related studies of reproductive toxicity. Then, at 30, 60, 90 and 120 days of age five pups were randomly selected, weighed and under chloroform (Merk Co.) inhalation anesthesia, their left and right ovaries were removed, trimmed of fat and extraneous tissue, weighed and fixed by immersion in Bouin’s solution for 24 hours.

### Microscopic study

Following tissue processing, 5 μm serial paraffin sections were prepared and stained with hematoxylin- eosin. For microscopic analysis, sections were selected using a non-random 10% sampling. Numbers of ovarian follicles and corpora lutea were counted in each 10th section of the ovary (23), so that each counted section was separated by a distance of approximately 50-60 μm from the next 10th section. Differential follicle counting and categorizing was performed by a blinded person. Ovarian follicles were classified on the basis of ovarian follicle morphology. Follicles that contained a single layer of squamous follicular cells were considered as primordial; the primary follicle contains an oocyte surrounded by a single layer of cuboidal follicular cells; the secondary follicle contains more than one layer of follicular cells around the oocyte and the antrum was not present; and the follicles containing scattered spaces or a distinct antrum were considered as antral (24). All follicles counted were classified as either healthy or atretic, respectively; according to the absence or presence of signs of oocyte and/or granular degeneration, such as pyknosis of the nucleus and infolding of the cell wall in the oocyte, ingression of granulosa cells within the antral cavity, pulling away of granulosa cells from the basement membrane, infolding and thickening of base membrane and uneven layers of granulosa cells (25).

For measuring the diameter of ovarian follicles in each developmental stage, 45 microscopic fields were randomly chosen in each rat. Then, using an ocular micrometer of light microscopy (Olympus EH), at a magnification of ×10, the largest and smallest diameters of each ovarian follicle were measured and the mean was calculated. To avoid counting the same follicle more than once, only individual follicles having an oocyte with a nucleus were evaluated, and we measured the size of the follicles in which the oocyte was present with an ocular micrometer.

### Statistical analysis

All data were analyzed using SPSS version 10.0 for Windows. The data in different groups were compared by one-way analysis of variance (ANOVA) and Tukey’s test was used as a post hoc test. Differences were considered to be significant when p<0.05, p<0.01 and p<0.001.

## Results

Mean body weight showed significant decreases in the highest dose group at 30 (p<0.001), 60 (p<0.01) and 90 and 120 (p<0.05) days of age in comparison with control group. Significant (p<0.05) decreases were observed in the moderate dose group at 30 days of age in comparison with the control group (Table 1).

There were significant differences between mean relative ovary weight in the 300 mg/L/day dose group and control group at 30, 60 and 90 (p<0.05) days of postnatal development (Table 1). No statistically significant differences were seen between mean relative ovary weight in the 20 and 100 mg/L/day dose groups at different stages of postnatal development.

Mean number of primordial follicles was higher significantly at 30 days of age in 100 (p<0.01) and 300 (p<0.001) mg/L/day dose groups and at 60 (p<0.01) and 90 (p<0.05) days of age in the 300 mg/L/day dose group in comparison with the control group (Table 2).

**Table 1:** Mean ± SEM body weight (g) and relative ovary weight (%) in control and neonatal lead-treated offspring Wistar rats during different stages of postnatal development


Groups	Days of age	Body weight	Relativeovary weight

**Control (a)**	30	28.37 ± 0.71^c^^d^	0.055 ± 0.003^d^
	60	79.96 ± 1.62^d^	0.046 ± 0.001^d^
	90	84.38 ± 2.13^d^	0.054 ± 0.002^d^
	120	95.12 ± 2.16^d^	0.050 ± 0.002
**20 mg/L/day(b)**	30	24.54 ± 0.18^d^	0.053 ± 0.003
	60	77.91 ± 1.27^d^	0.045 ± 0.003
	90	81.65 ± 2.38^d^	0.051 ± 0.002
	120	91.65 ± 2.01	0.050 ± 0.001
**100 mg/L/day(c)**	30	20.47 ± 0.35^a^^*^^*^	0.052 ± 0.002
	60	74.66 ± 2.80	0.043 ± 0.001
	90	79.25 ± 2.81	0.053 ± 0.001
	120	91.05 ± 2.11	0.048 ± 0.003
**300 mg/L/day(d)**	30	17.26 ± 1.50^a^^b^^*^^*^^*^	0.050 ± 0.002^a^^*^
	60	69.29 ± 1.24^a^^b^^*^^*^	0.040 ± 0.002^a^^*^
	90	75.82 ± 1.16^a^^b^^*^	0.047 ± 0.003^a^^*^
	120	85.42 ± 2.36^a^^*^	0.048 ± 0.001


Different letters indicates significant (p<0.05) differences between groups. *Significant difference between control and treatment groups. *p<0.05, **p<0.01 and ***p<0.001

**Table 2:** Mean ± SEM number of ovarian follicles in control and neonatal lead-treated offspring Wistar rats during different stages of postnatal development


Groups	Days of age	Primordial F.	Primary F.	Secondary F.	Antral F.

**Control(a)**	30	12.30 ± 0.27^c^^d^	17.30 ± 0.25^c^^d^	17.20 ± 0.35^c^^d^	6.63 ± 0.41^c^^d^
	60	12.07 ± 0.23^c^	16.97 ± 0.21^d^	15.80 ± 0.48d	6.93 ± 0.47^d^
	90	11.97 ± 0.15^d^	16.55 ± 0.38^d^	15.87 ± 0.25^b^^d^	6.97 ± 0.46^d^
	120	11.32 ± 0.20	15.43 ± 0.23	16.64 ± 0.45	7.65 ± 0.50
**20 mg/L/day(b)**	30	12.36 ± 0.25^c^	17.11 ± 0.38^d^	16.92 ± 0.22^d^	5.39 ± 0.15^d^
	60	12.63 ± 0.21	16.56 ± 0.27	15.44 ± 0.28	6.58 ± 0.23^d^
	90	12.08 ± 0.18	16.35 ± 0.31	15.49 ± 0.33	6.17 ± 0.21^d^
	120	11.47 ± 0.20	15.30 ± 0.24	15.55 ± 0.28	6.71 ± 0.20
**100mg/L/day(c)**	30	14.87 ± 0.36^a^^*^^*^	13.03 ± 0.21^a^^*^	13.79 ± 0.42^a^^*^	4.67 ± 0.22^a^^*^
	60	12.79 ± 0.28	15.34 ± 0.32	14.13 ± 0.45	4.03 ± 0.31^a^^*^
	90	12.10 ± 0.16	16.20 ± 0.62	15.23 ± 0.60	5.83 ± 0.26
	120	11.65 ± 0.24	15.05 ± 0.51	15.84 ± 0.46	6.46 ± 0.19
**300mg/L/day(d)**	30	15.63 ± 0.26^a^^*^^*^^*^	12.53 ± 0.30^a^^b^^*^^*^	11.80 ± 0.38^a^^b^^*^^*^	3.4 ± 0.20^a^^b^^*^^*^
	60	15.52 ± 0.42^a^^*^^*^	13.12 ± 0.31^a^^*^	12.31 ± 0.43^a^^*^	4.86 ± 0.31^a^^b^^*^
	90	13.07 ± 0.32^a^^*^	15.01 ± 0.55	14.73 ± 0.54	5.50 ± 0.28
	120	12.11 ± 0.29	15.21 ± 0.34	15.33 ± 0.50	6.00 ± 0.25


Different letters indicates significant (p<0.05) differences between groups. *Significant difference between control and treatment groups. *p<0.05, **p<0.01 and ***p<0.001

**Table 3:** Mean (±SEM) number of ovarian follicles in control and neonatal lead-treated offspring Wistar rats during different stages of postnatal development


Groups	Days of age	Primordial F.	Primary F.	Secondary F.	Antral F.

**Control(a)**	30	21.40 ± 0.66	52.61 ± 0.55	99.40 ± 4.60^c^^d^	207.17 ± 4.05^c^^d^
	60	22.93 ± 0.75	52.57 ± 0.75	103.00 ± 2.26^d^	232.00 ± 3.34^c^^d^
	90	22.50 ± 0.74	52.57 ± 0.69	106.87 ± 3.27^d^	237.00 ± 5.23^d^
	120	21.42 ± 0.80	52.56 ± 0.54	108.33 ± 3.30	244.54 ± 4.14
**20 mg/L/day(b)**	30	21.30 ± 0.37	52.30 ± 0.51	98.10 ± 2.25^d^	205.36 ± 3.14^d^
	60	22.57 ± 0.46	51.92 ± 0.63	100.33 ± 1.88	225.97 ± 2.20^d^
	90	21.74 ± 0.39	51.87 ± 0.41	102.71 ± 2.38	231.57 ± 3.32^d^
	120	21.56 ± 0.60	52.66 ± 0.49	107.22 ± 1.80	240.54 ± 2.77
**100mg/L/day(c)**	30	21.13 ± 0.64	52.34 ± 0.46	90.16 ± 4.53^a^^*^	193.50 ± 4.41^a^^*^
	60	22.90 ± 0.74	52.11 ± 0.71	97.67 ± 1.82	217.33 ± 6.63^a^^*^
	90	22.45 ± 0.73	51.76 ± 0.52	100.67 ± 3.70	229.67 ± 9.83
	120	22.31 ± 0.61	52.08 ± 0.48	104.77 ± 2.47	237.38 ± 5.11
**300mg/L/day(d)**	30	20.27 ± 0.44	52.18 ± 0.54	87.83 ± 2.90^a^^b^^*^^*^	188.15 ± 2.89^a^^b^^*^^*^
	60	22.68 ± 0.52	51.69 ± 0.33	96.03 ± 2.50^a^^*^	209.63 ± 2.10^a^^b^^*^^*^
	90	21.86 ± 0.65	51.55 ± 0.52	98.33 ± 3.29^a^^b^^*^	222.33 ± 2.34^a^^b^^*^
	120	22.05 ± 0.58	51.81 ± 0.50	102.36 ± 2.44	235.81 ± 2.02


Different letters indicates significant (p<0.05) differences between groups. * Significant difference between control and treatment groups. *p<0.5, **p<0.01, ***p<0.001

**Fig 1:**
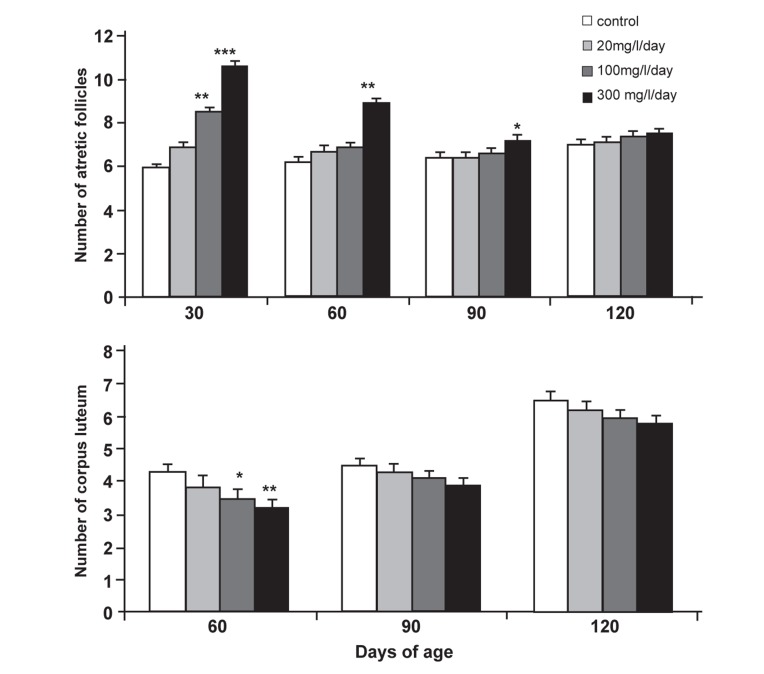
Comparison of mean ± SEM number of atretic follicles and corpus luteum in control and neonatal lead-treated offspring Wistar rats during different stages of postnatal development. *p<0.05, **p<0.01 and ***p<0.001.

**Fig 2:**
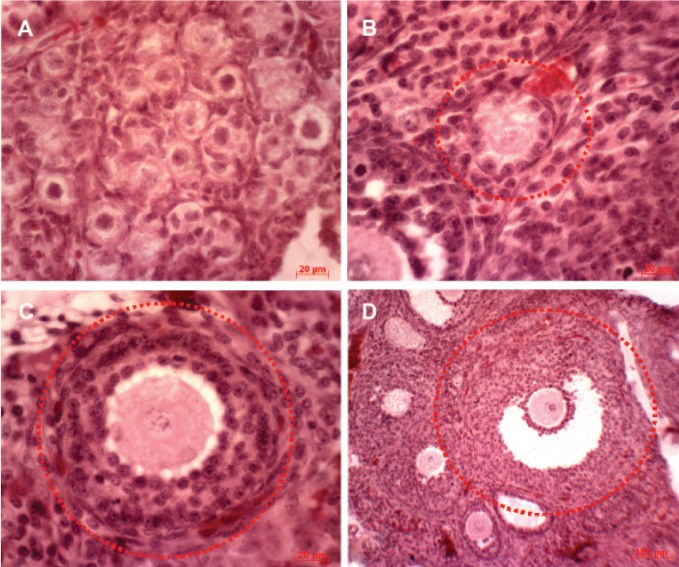
Histological sections of ovarian follicles in ovary of offspring Wistar rats at 60 days of age in control group (hematoxyline & eosin); primordial (A) (scale bar: 20 μm), primary (B) (scale bar: 100 μm), secondary (C) (scale bar: 100 μm) and antral (D) (scale bar: 100 μm) follicles.

**Fig 3:**
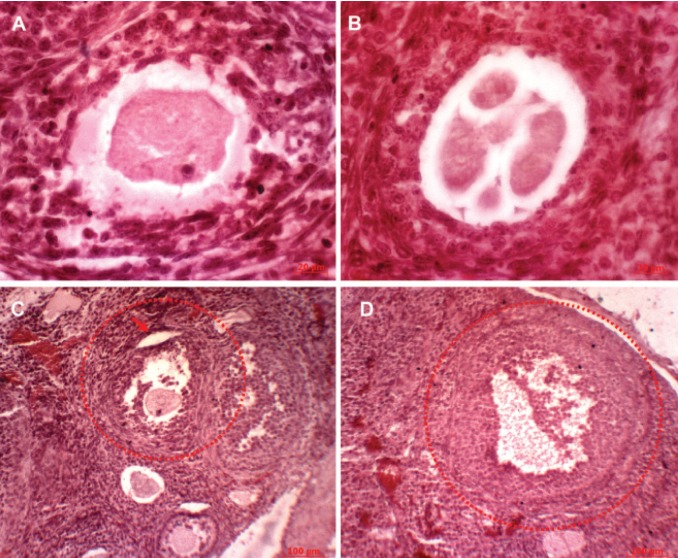
Histological sections of ovarian follicles in ovary of offspring Wistar rats at 60 days of age in control group (hematoxyline & eosin); primordial (A) (scale bar: 20 μm), primary (B) (scale bar: 100 μm), secondary (C) (scale bar: 100 μm) and antral (D) (scale bar: 100 μm) follicles.

Significant decreases were observed in the mean numbers of primary, secondary and antral follicles at 30 days of age in 100 (p<0.05) and 300 (p<0.01) mg/L/day dose groups and at 60 (p<0.05) days of age in the 300 mg/L/day dose group in comparison with the control group (Table 2). There was no significant difference between the mean numbers of ovarian follicles in the 20 mg/L/day dose group and control group at different stages of postnatal development.

In addition, the means of secondary and antral follicle diameters decreased significantly (p<0.05) in the 100 mg/L/day dose group at 30 (p<0.05) days of age and in the 300 mg/L/day dose group at 30 (p<0.01), 60 and 90 (p<0.05) days of age in comparison with the control group (Table 3).

There were significant increases in the mean number of atretic follicles at 30 days of age in 100 (p<0.01) and 300 (p<0.001) mg/L/day dose groups and at 60 (p<0.01) and 90 (p<0.05) days of postnatal development in the 300 mg/L/day dose group in comparison with the control group (Figes1, 2 and 3).

Significant decreases were seen in the mean number of corpora lutea in 100 (p<0.05) and 300 (p<0.01) mg/L/day dose groups at 60 days of age in comparison with the control group (Fig 1).

## Discussion

During recent decades concerns have been raised about human infertility that might stem from exposure to environmental contamination. Exposure to environmental contamination prior and after the initiation of pregnancy, and during the early period of postnatal development could affect reproductive efficacy of offspring (26). Although few studies have been performed in women, several cases of lead poisoning have been associated with sterility, miscarriage, abortion, premature delivery and infant mortality (27, 28). The present study showed that maternal lead acetate exposure affects prepubertal ovarian follicle development in a dose-related manner and can reduce fertility and reproductive efficiency of offspring Wistar rats.

Mean body weight of the offspring decreased significantly in neonatal lead treatment of Wistar rats, particularly in the 300 mg/L/day dose group. Ronis et al. observed that lead exposure during pregnancy and lactation resulted in significant dose-responsive decreases in birth weight and crown-to-rump length in all litters of the treatment group (20). Cezard and Haguenoer reported that lead intoxication resulted in body weight reduction caused by a loss of appetite (29).

Neonatal lead treatment caused dose-related reductions of ovaries’ relative weights in offspring rats. It seems that these reductions may be due to dosedependent increases of atretic follicles, as well as decreases of secondary and antral follicular diameters in the ovaries of rats’ offspring. McGivern et al. and El Feki et al. have shown that ovarian weight reduces in offspring exposed maternally to low levels of lead (30, 31). Mansouri has reported that lead treatment causes follicular atresia, reduction of tertiary follicle size, loosening of junctions between granulosa cells and destruction and degeneration of oocytes (32). Azarnia et al. found that the number of atretic follicles increased significantly (p<0.05) in NMRI mice exposed to lead acetate at a dose of 10 mg/kg/week for 15 weeks (33).

The present study showed that neonatal lead treatment reduced the number of primary, secondary and antral follicles in the ovaries of offspring rats, particularly in the 300 mg/L/day dose group. Junaid et al. showed that low lead acetate levels reduced small and medium follicle numbers and high levels resulted in fewer large follicles numbers in mice (34). Dose-dependent reductions of growing follicles and the presence of higher numbers of primordial follicles suggest that neonatal lead treatment inhibits transition from the primordial to primary follicle stage. In addition, neonatal lead treatment causes a reduction in number of corpora lutea in the ovaries of offspring rats at puberty.

McGivern et al. and El-Feki et al. found that maternal exposure to low lead levels causes fewer corpora lutea and abnormal estrous cycles in offspring (30, 31). Also, a significant decrease in serum progesterone levels was seen in female Rhesus monkeys exposed to lead acetate for 75 months via drinking water, indicating that luteal function was blocked by lead (13).

Overall, these data suggest that neonatal lead treatment inhibits follicular development in the ovaries of offspring in a dose-related manner. Ercal et al. observed that chronic exposure to lead damaged primordial and medium follicles and arrested follicular development in Rhesus monkeys (35). Crystel et al. showed that even low doses of lead provoked an inhibition in folliculogenesis leading to dysfunction of this process (36). It has been reported that lead acetate reduces the number of primordial follicles and increases atretic antral follicle number in mice (5). In rats the formation of primordial follicles is completed by around postnatal day 3 or 4 (37). During postnatal development some of primordial follicles grew and primary, preantral and antral follicles were seen in the ovaries from 9 to 20 days of age (38). The first estrus and ovulation occured between 35-42 days of age (39).

However, the present study showed no significant differences in numbers of growing follicles and corpora lutea at 90 and 120 days of age in the treatment groups. Additionally, the mean numbers of secondary and antral follicles, and ovarian weight in the treatment groups normalized until 120 days of age in comparison with 30 days of age. In this regard, Mansouri and Abdennour have shown that increase of exposure time to lead caused more toxic effects to gametes (32). It seems that lead has transient effects on follicular development in the ovary of offspring and ovarian parameters become better gradually until 120 days of age. Thus, our results show the reversibility of toxic effects of neonatal lead treatment on the follicular development in ovaries of offspring rats. Also, Piasek and Kostial concluded that the adverse reproductive action of lead is reversible after withdrawal of adult female Albino rats from exposure (40).

## Conclusion

Consequently, the present study shows that maternal lead acetate exposure during lactation affects prepubertal ovarian follicle development in a dose dependent manner, but ovarian parameters become better gradually during the postpubertal period.

## References

[1] Hruska KS, Furth PA, Seifer DB, Sharara FI, Flaws JA (2000). Environmental factors in infertility. Clin Obstet Gynecol.

[2] Hoyer PB (2005). Damage to ovarian development and function. Cell Tissue Res.

[3] Goyer RA (1989). Mechanism of lead and cadmium nephrotoxicity. Toxicol Lett.

[4] Dearth RK, Hiney JK, Srivastava V, Burdick SB, Bratton GR, Dees WL (2002). Effects of lead (Pb) exposure during gestation and lactation on female pubertal development. Reprod Toxicol.

[5] Taupeau C, Poupon J, Nome F, Lefevre B (2001). Lead accumulation in the mouse ovary after treatment-induced follicular atresia. Reprod Toxicol.

[6] Dietrich KN (1991). Human fetal lead exposure: Intrauterine growth, maturation and postnatal development. Fundam Appl Toxicol.

[7] Battacharayya MH (1983). Bioavailability of orally administered cadmium and lead to the mother, fetus and neonate during pregnancy and lactation: An overview. Sci Total Environ.

[8] Golmohammadi T, Ansari M, Nikzamir AR, Safary Abhari R, Elahi S (2007). The effect of maternal and fetal lead concentration on birth weight: polluted versus non-polluted areas of Iran. TUMJ.

[9] Mansoori M, Shah Farhat A, Mohammadzadeh A (2009). The evaluation of the effect of maternal blood lead concentration on the incidence of delivery of low birth weight neonates. Scientific Journal of Kurdistan University of Medical Sciences.

[10] Coogan TP, Shiraishi N, Waalkes MP (1994). Apparent quiescence of the metallothione in gene in rat ventral prostate: Association with cadmium-induced prostate tumors in rats. Environ Health Perspect.

[11] Gerber GB, Leonard A, Jacquet P (1980). Toxicity, mutagenicity and teratogenicity of lead. Mutat Res.

[12] Avazeri N, Denys A, Lefèvre B (2006). Lead cations affect the control of both meiosis arrest and meiosis resumption of the mouse oocyte in vitro at least via the PKC pathway. Biochimie.

[13] Junaid M, Chowdhuri DK, Narayan R, Shanker R, Saxena DK (1997). Lead-induced changes in ovarian follicular development and maturation in mice. J Toxicol Environ Health.

[14] Francks PA, Laughlin NK, Dierschke DJ, Bowman RE, Meller PA (1989). Effects of lead on luteal function in Rhesus Monkey. Biol Reprod.

[15] Hilderbrand DC, Der R, Griffin WT, Fahim MS (1973). Effect of Lead acetate on reproduction. Am J Obstet Gynecol.

[16] Stowe HD, Goyer RA (1971). The reproductive ability and progeny of F1 lead-toxic rats. Fertil Steril.

[17] Laughlin NK, Bowman RE, Franks PA, Dierschke DJ (1987). Altered menstrual cycles in Rhesus monkeys induced by lead. Fundam App Toxicol.

[18] Ronis MJ, Badger TM, Shema SJ, Roberson PK, Shaikh F (1996). Reproductive toxicity and growth effects in rats exposed to lead at different periods during development. Toxicol Appl Pharmacol.

[19] Ronis MJ, Badger TM, Shema SJ, Roberson PK, Templer L, Ringer D (1998). Endocrine mechanisms underlying the growth effects of developmental lead exposure in the rat. J Toxicol Environ Health A.

[20] Coffigny H, Thoreux-Manalay A, Pinon-Lataillade G, Monchaux G, Masse R, Soufir JC (1994). Effects of lead poisoning of rats during pregnancy on the reproductive system and fertility of their offspring. Hum Exp Toxicol.

[21] Ronis MJ, Badger TM, Shema SJ, Roberson PK, Shaikh F (1998). Effects on pubertal growth and reproduction in rats exposed to lead perinatally or continuously throughout development. J Toxicol Environ Health A.

[22] Ronis MJ, Shahare M, Mercado C, Irby D, Badger TM (1994). Disrupted reproductive physiology and pubertal growth in rats exposed to lead during different developmental periods. Biol Reprod.

[23] Bolon B, Bucci TJ, Warbritton AR, Chen JJ, Mattison DR, Heindel JJ (1997). Differential follicle counts as a screen for chemically induced ovarian toxicity in mice: results from continuous breeding bioassays. Fundam Appl Toxicol.

[24] Britt KL, Drummond AE, Cox VA, Dyson M, Wreford NG, Jones ME (2000). An age-related ovarian phenotype in mice with targeted disruption of the Cyp 19 (aromatase) gene. Endocrinology.

[25] Myers M, Britt KL, Wreford NG, Ebling FJP, Kerr JB (2004). Methods for quantifying follicle numbers within the mouse ovary. Reproduction.

[26] Michal F, Grigor KM, Negro-Vilar A, Skakkebak NE (1993). Impact of the environment on reproductive health: executive summary. Environ Health Perspect.

[27] Gerhard I, Waibel S, Daniel V, Runnebaum B (1998). Impact of heavy metals on hormonal and immunological factors in women with repeated miscarriages. Hum Reprod Update.

[28] Winder C (1993). Lead, reproduction and development. Neurotoxicology.

[29] Cezard C, Haguenoer JM (1992). Toxicologie du plomb chez l’homme.Technique et documentation.

[30] McGivern RF, Sokol RZ, Berman NG (1991). Prenatal lead exposure in the rat during the third week of gestation: long-term behavioral, physiological,, and anatomical effects associated with reproduction. Toxicol Appl Pharmacol.

[31] El-Feki A, Ghorbel F, Smaoul M, Makni-Ayadi F, Kammoun A (2000). Effects of cars lead on the general growth and sexual activity in rats. Gynecol Obstet Fertil.

[32] Mansouri O, Abdennour A (2008). Influence of sudden cystine supplementation and suppression on adrenal and ovary of lead exposed rat. European Journal of Scientific Research.

[33] Azarnia M, Shakour A, Rostami P, Sanaie-Mehr A (2004). The protective role of L-Cysteine against follicular atresia induced by lead in mouse ovary. Acta Medica Iranica.

[34] Junaid M, Chowdhuri DK, Narayan R, Shanker R, Saxena DK (1997). Lead-induced changes in ovarian follicular development and maturation in mice. J Toxicol Environ Health.

[35] Ercal N, Treeratphan P, Lutz P, Hammond TC, Matthews RH (1996). N-actylcysteine protects Chinese hamster ovary (CHO) cells from lead induced oxidative stress. Toxicology.

[36] Taupeau C, Poupon J, Nomé F, Lefèvre B (2001). Lead accumulation in the mouse ovary after treatment-induced follicular atresia. Reprod Toxicol.

[37] Kezele P, Skinner MK (2003). Regulation of ovarian primordial follicle assembly and development by estrogen and progesterone: endocrine model of follicle assembly. Endocrinology.

[38] Hirshfield AN (1991). Development of follicles in the mammalian ovary. Int Rev Cytol.

[39] Rennels EG (1951). Influence of hormones on the histochemistry of ovarian interstitial tissue in the immature rat. Am J Anat.

[40] Piasek M, Kostial K (1991). Reversibility of the effects of lead on the reproductive performance of female rats. Reprod Toxicol.

